# PECAM1^+^/Sca1^+^/CD38^+^ Vascular Cells Transform into Myofibroblast-Like Cells in Skin Wound Repair

**DOI:** 10.1371/journal.pone.0053262

**Published:** 2013-01-04

**Authors:** Julia Etich, Vera Bergmeier, Christian Frie, Sandra Kreft, Lena Bengestrate, Sabine Eming, Cornelia Mauch, Beate Eckes, Hikmet Ulus, Frances E. Lund, Gunter Rappl, Hinrich Abken, Mats Paulsson, Bent Brachvogel

**Affiliations:** 1 Center for Biochemistry, Medical Faculty, University of Cologne, Cologne, North Rhine-Westphalia, Germany; 2 Center for Molecular Medicine Cologne (CMMC), Medical Faculty, University of Cologne, Cologne, North Rhine-Westphalia, Germany; 3 Department of Dermatology, University of Cologne, Cologne, North Rhine-Westphalia, Germany; 4 Clinic for Paediatric Surgery, Cologne, North Rhine-Westphalia, Germany; 5 Department of Medicine, University of Rochester Medical Center, Rochester, New York, United States of America; 6 Tumorgenetics, Department I of Internal Medicine, Medical Faculty, University of Cologne, Cologne, North Rhine-Westphalia, Germany; 7 Cologne Excellence Cluster on Cellular Stress Responses in Aging - Associated Diseases (CECAD), Cologne, North Rhine-Westphalia, Germany; University of Maryland School of Medicine, United States of America

## Abstract

Skin injury induces the formation of new blood vessels by activating the vasculature in order to restore tissue homeostasis. Vascular cells may also differentiate into matrix-secreting contractile myofibroblasts to promote wound closure. Here, we characterize a PECAM1^+^/Sca1^+^ vascular cell population in mouse skin, which is highly enriched in wounds at the peak of neoangiogenesis and myofibroblast formation. These cells express endothelial and perivascular markers and present the receptor CD38 on their surface. PECAM1^+^/Sca1^+^/CD38^+^ cells proliferate upon wounding and could give rise to α-SMA^+^ myofibroblast-like cells. CD38 stimulation in immunodeficient mice reduced the wound size at the peak of neoangiogenesis and myofibroblast formation. In humans a corresponding cell population was identified, which was enriched in sprouting vessels of basal cell carcinoma biopsies. The results indicate that PECAM1^+^/Sca1^+^/CD38^+^ vascular cells could proliferate and differentiate into myofibroblast-like cells in wound repair. Moreover, CD38 signaling modulates PECAM1^+^/Sca1^+^/CD38^+^ cell activation in the healing process implying CD38 as a target for anti-angiogenic therapies in human basal cell carcinoma.

## Introduction

The interaction of fibroblasts and vascular cells with the microenvironment is crucial to restore tissue integrity in skin wound healing. Resident fibroblasts are activated upon tissue injury to repopulate the wounded area and reconstruct the connective tissue. Therefore, fibroblasts undergo significant phenotypic changes into migrating and extracellular matrix-secreting myofibroblasts. Changes in the wound environment also initiate an angiogenic response during wound repair. Lining endothelial and perivascular cells migrate into the wound and form a new vascular bed to facilitate an adequate oxygen and nutrient supply. Both cell types may also transform into myofibroblast-like cells to promote non-vessel tissue repair [1]. Previous experiments indicated that PECAM1^+^ endothelial cells can adapt a myofibroblast-like phenotype by forming α-smooth muscle actin (α-SMA)-containing stress fibers in corneal wounds [2] and give rise to fibroblast-like cells [3]. Perivascular cells (PVCs) are assumed to be activated during skin wound healing to migrate to the perivascular space and transform into myofibroblast-like cells [4], [5]. Moreover, PVCs display mesenchymal stem cell-like properties [6], [7] and are thought to contribute to the fibrotic reactions in spinal cord scar tissue formation [8]. Hence, endothelial cells and perivascular cells could represent a source for mesenchymal cells upon tissue repair [9], [10]. Sca1 is often used to identify subpopulation of endothelial progenitor cells in the bone marrow or in the circulation [11], [12]. We have previously detected Sca1 at the cell surface of a perivascular cell population in the vasculature of adult brain meninges [7], [13] that can differentiate into various mesenchymal cell types.

In this work, we have used the expression of Sca1 and PECAM1 to analyze the contribution of the vasculature to myofibroblast formation and wound repair in the skin [9], [10]. We identified a vascular PECAM1^+^/Sca1^+^ cell subpopulation, which was highly enriched in the granulation tissue of skin wounds and in neoangiogenic areas of human basal cell carcinoma. Surprisingly, cells expressed perivascular cell-specific genes like *Desmin*, *Pdgfrb* and *Angp1* and the endothelial cell-specific genes *Angpt2* and *Tie2.* In addition, the PECAM1 receptor *CD38* was found exclusively in PECAM1^+^/Sca1^+^ cells. PECAM1^+^/Sca1^+^/CD38^+^ cells entered the cell cycle upon wounding and sorted cells could give rise to myofibroblast-like cells. Gain-of-function experiments in mice indicated a relevance of the CD38 receptor signaling for the activation of PECAM1^+^/Sca1^+^ cells in skin repair. Since CD38 can regulate cell activation, adhesion and migration events and its expression is restricted to PECAM1^+^/Sca1^+^ cells in the wound we propose that PECAM1^+^/Sca1^+^ cells can be activated by CD38 to transform into myofibroblast-like cells during wound repair. Data moreover imply CD38 as a target for anti-angiogenic therapy of human basal cell carcinoma.

## Materials and Methods

### Ethics Statement, Mice, Biopsies

Animal experiments were performed with C57BL/6N or C.129S6(B6)-Rag2tm1FwaN12 mice on BALB/c background in accordance with the animal ethics guidelines of the German law. Institutional review board: “Landesamt für Natur, Umwelt- und Verbraucherschutz NRW”, (ethic approval no. 31.08.261, 20.11.057). Human foreskin samples and basal cell carcinoma biopsies were provided by the SFB829-core facilities. Institutional review board:” Ethik-Kommission der Medizinischen Fakultät der Universität zu Köln” (Ethic approval no. 08-144). Written informed consent was obtained from the patients.

### Histology and Immunohistochemistry

Cryosections were used for histological and immunohistochemical analysis. For morphological assessment sections were stained for nuclei (hematoxylin) and cytoplasm (eosin) according to standard procedures. For immunofluorescence studies monoclonal rat anti-CD31 (BD), mouse anti- α-SMA (Sigma), mouse anti-desmin (Progen), rat anti-Sca1 (BD), rat anti-CD38 (Biolegend), rat anti-Tie2 (BD), rat anti-CD34 (BD) antibodies were used on paraformaldehyde-fixed sections. Corresponding secondary antibodies coupled to Alexa Fluor 488 (Molecular Probes) or Cy3 (Jackson Immuno Research) and DAPI (Invitrogen) were applied. Images were taken and analyzed by immunofluorescence (Nikon Eclipse TE2000-U Microscope) and confocal microscopy (LeicaTCS-SP5).

### Wound Healing Experiments

Mice were anesthetized by i.p. injection of Ketavet/Rompun (Bayer) and full thickness wounds of 6 mm diameter were inflicted at the back. For antibody treatments 75 µg/150 µl of rat anti-CD38 or isotype control antibody (SouthernBiotech) were injected on day two, four and six post wounding into the retro-orbital venous sinus. Wounds were embedded in tissue tek (Sakura Finetek) and serial sections from the central portion of the wound (Leica Cryotome, CM3050) were stained with hematoxylin and eosin (H&E). Morphometric parameters [14] were determined using ImageJ software [15].

### Fluorescence Activated Cell Sorting and Cell Culture

Isolated skin or wounds were digested with 0.5% (w/v) Dispase® II (Roche) in PBS at 4°C overnight. Subcutaneous tissue and epidermis were removed, dermis incubated in 0.2% (w/v) collagenase type 1 (Worthington) in DMEM-F12, 10% FCS for 1 ½ h at 37°C, washed with 5% FCS/PBS and passed through a 40 µm cell strainer (BD). ∼6×10^5^ cells were recovered from skin or wound of a mouse. Cells were incubated for 15 min on ice in 5% FCS/PBS with mouse-Fc-Block (BD), and then stained for 15 min on ice with primary conjugated monoclonal antibodies specific for CD31, CD34, CD38, CD45, Sca1 or Tie2. Cells were washed, costained with 7-actinomycin (7AAD) followed by flow cytometry (FACSCantoII, BD) or cell sorting (FACSAriaIII, BD). To detect intracellular α-SMA, cells were fixed and permeabilized in IntraSure solution (BD) prior staining. For cell cycle analysis cells were labeled with antibodies, fixed and permeabilized in IntraSure solution (BD) and costained with propidium iodide (BD). Cells were analyzed by flow cytometry. The Watson algorithm [16] of the Flow Jo software (Tree Star, Inc) was used for data processing. For cell culture experiments 5000 sorted cells were cultured in VascuLife SMC medium (LifeLine Technology) containing 5 ng/ml FGF, EGF, insulin, 50 ng/ml ascorbic acid and 5% FCS at 37°C with 5% CO_2_.

### Semiquantitative RT-PCR

RNA from sorted cells was isolated using NucleoSpin-RNA-XS Kit (Macherey&Nagel) and quality was assessed by capillary electrophoresis according to the manufacturer’s specifications (Agilent2100Bioanalyzer). RNA samples were reversely transcribed into cDNA using the SuperscriptIII 1°-strand Synthesis Kit (Invitrogen). Semiquantitative RT-PCRs were performed for *Desmin*, *Pdgfrb*, *Angpt1*, *Angpt2*, *Tie2*, *Pecam1*, *Sca1*, *CD34*, *CD38* and *Gapdh* according to standard procedures and band intensities of the electrophoretic gels were processed and quantified using Image J software [15]. Primer sequences will be provided on request.

## Results

### The Vasculature is Reorganized During Skin Repair

**Figure 1:**
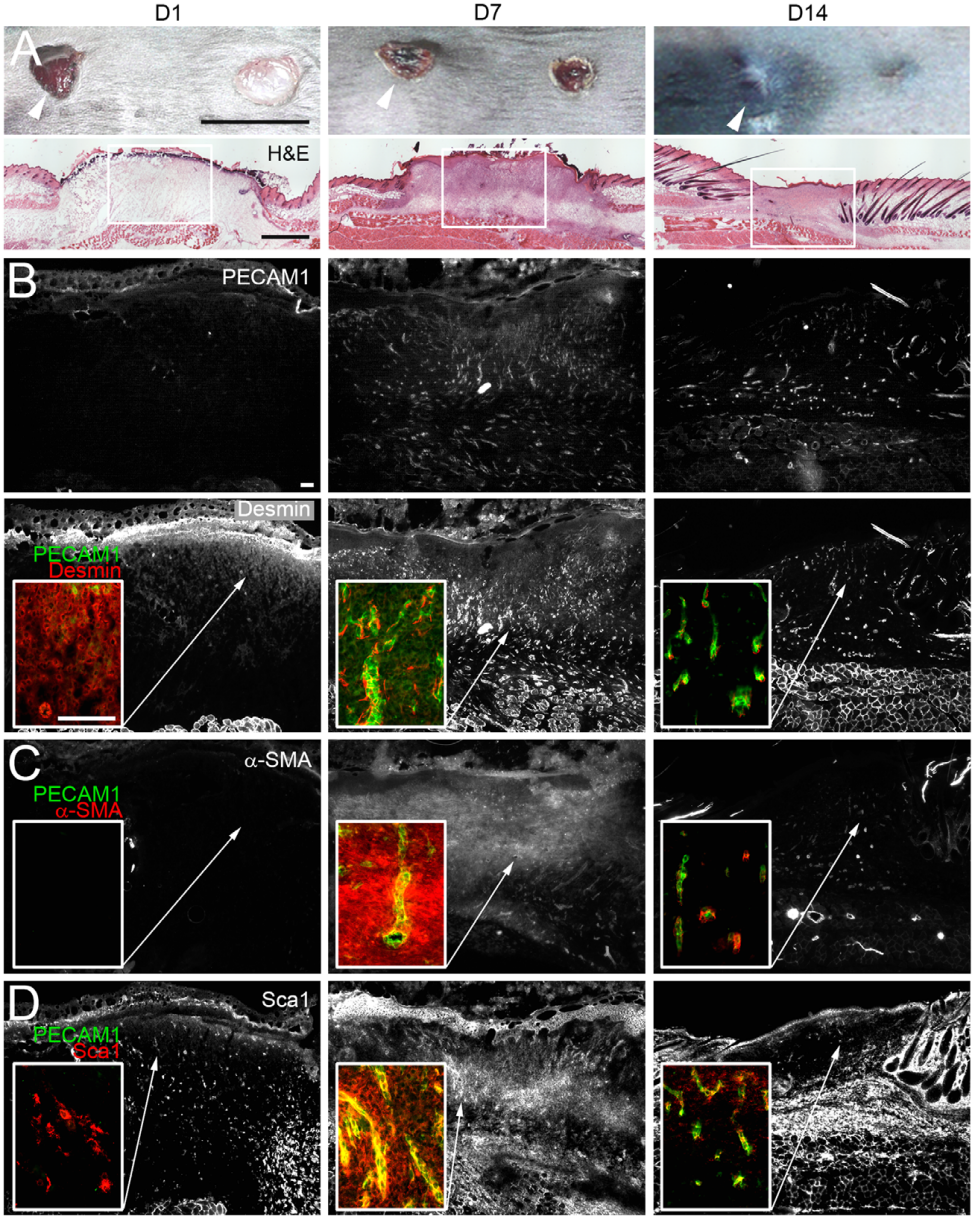
Expression of perivascular and endothelial cell-specific markers in wound repair. Analysis of PECAM1, desmin, α-SMA and Sca1 expression in the wounded skin. (A) Dorsal view of full thickness wounds on the back skin of mice one (D1), seven (D7) and 14 (D14) days post injury. Representative H&E-stained cryosections of selected wounds (arrowhead) during inflammation (D1), granulation (D7) and remodeling (D14) are shown. (B-D) Immunostaining of (B) PECAM1/desmin, (C) PECAM1/α-SMA or (D) PECAM1/Sca1 expression at the different stages of wound healing. The individual monochrome signals for PECAM1, desmin, α-SMA and Sca1 are shown in overviews. Squares within the images represent closeups of overlays for the PECAM1/desmin, PECAM1/α-SMA PECAM1/Sca1 stainings (B-D). Bars 1 cm (A, top), 1 mm (A, lower panel), 100 µm (B).

To study the distribution of the vasculature in skin repair, wounds were inflicted at the back of eight weeks old C57BL/6N mice (Fig. 1A) and vascular marker expression was analyzed. In the early phase of inflammation hardly any PECAM1^+^ endothelial or desmin^+^ perivascular cells were seen in the wound (Fig. 1B). At seven days post wounding PECAM1^+^ endothelial and desmin^+^ perivascular cells had invaded the wound and desmin^+^ perivascular cells formed cytoplasmic protrusions along the PECAM1^+^ vessels. With vessel regression the number of PECAM1^+^/desmin^+^ cells decreased. α-SMA^+^ myofibroblasts were mainly detected in the granulation tissue seven days post injury (Fig. 1C). Stem cell antigen 1 (Sca1), known to be expressed in isolated perivascular cells from brain meninges [13], was hardly detected in the wound at one day post injury (Fig. 1D). After seven days the numbers of Sca1^+^ cells transiently increased in the stroma and vasculature of the granulation tissue, while in 14 days old wounds Sca1 expression was primarily detected in PECAM1^+^ vessels of the wound. Thus, the distribution of Sca1^+^ cells partially resembled that of desmin^+^ perivascular cells, PECAM1^+^ endothelial cells and α-SMA^+^ myofibroblasts.

### PECAM1^+^/Sca1^+^ Vascular Cells are Enriched During Wound Repair

**Figure 2:**
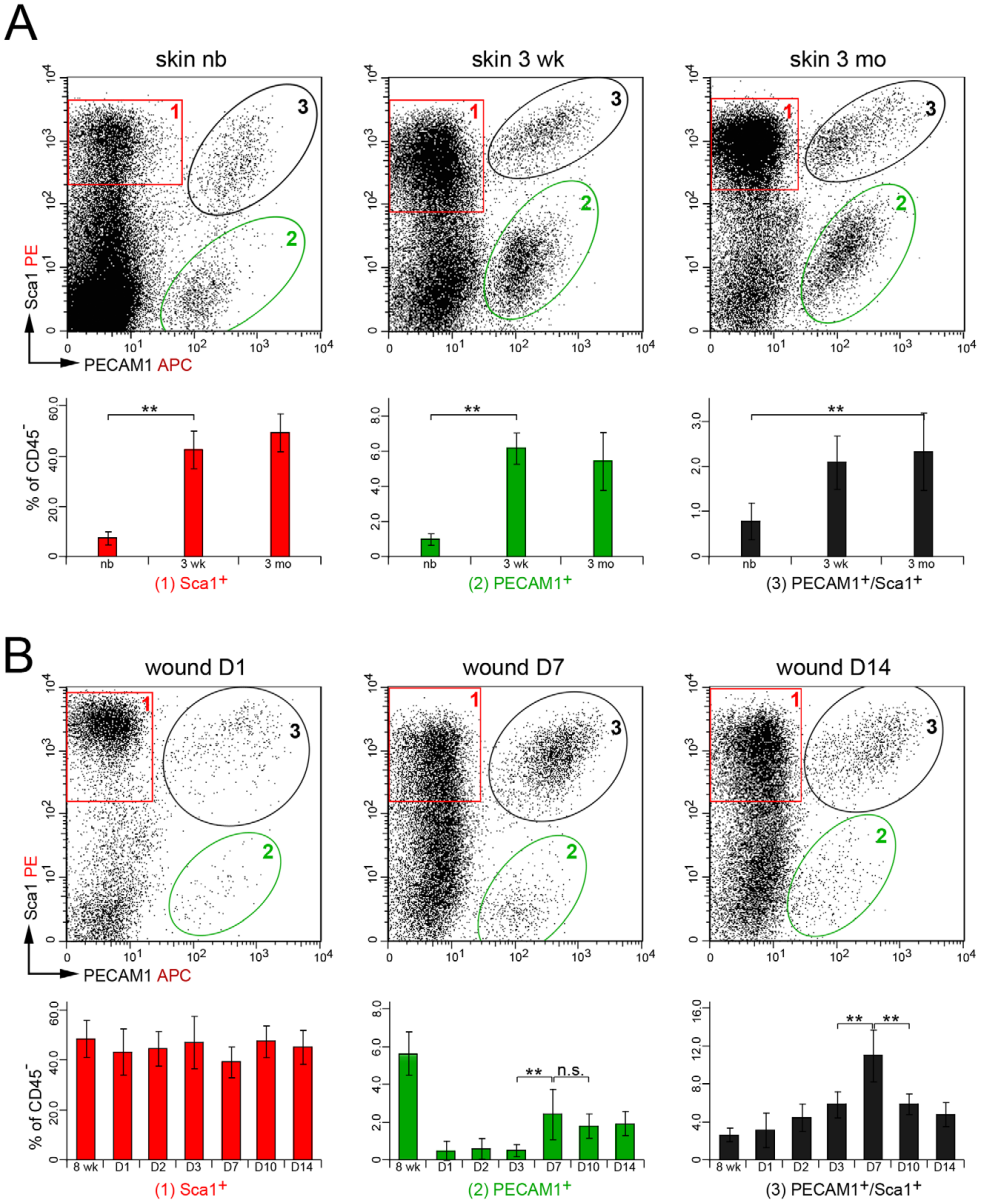
Distribution of PECAM1 and Sca1 protein on skin- and wound-derived cells. (A) Flow cytometry analysis of Sca1 and PECAM1 expression in CD45^-^ non-hematopoietic cells isolated from newborn (nb), three weeks (3 wk) and three months (3 mo) old dermis. Sca1^+^ (1, red box), PECAM1^+^ (2, green ellipse) and PECAM1^+^/Sca1^+^ (3, black ellipse) cell populations are highlighted. (B) Dot plots of Sca1 and PECAM1 expression in cell suspensions isolated from full thickness wounds one (D1), seven (D7) and 14 days (D14) post injury of eight weeks old mice. Percentage of positive cell populations at the different time points of flow cytometry analysis (lower panel) is given with standard deviation and significant changes were determined using the unpaired two-tailed student’s T-test (n≥3, **p≤0.01, n.s = not significant).

To define the origin of Sca1^+^ cells we analyzed cell suspensions from dermis and wounds at various times points using flow cytometry. Cells were stained for 7AAD and the cell surface markers CD45, Sca1 and PECAM1. In the newborn dermis Sca1^+^ cells represented 7.5% of the 7AAD^-^ viable CD45^-^ non-hematopoietic cell population, which increased to 43% and 49% in the juvenile and adult dermis, respectively (Fig. 2A, n≥10 mice). Approximately 1% of the newborn and up to 6.2% of the juvenile and adult dermal cells expressed PECAM1. In addition, 0.8% of the cells in newborn dermis and 2.3% in the juvenile and adult dermis expressed both proteins. In wounds no significant changes were seen for Sca1^+^ cells at the analyzed time points, whereas the proportion of PECAM1^+^ cells decreased from 5.6% in the unwounded control to 0.5% within the first three days after tissue injury (Fig. 2B, n≥4 mice). Later the proportion of PECAM1^+^ cells increased to 2.4% of the cells. The percentage of PECAM1^+^/Sca1^+^cells increased from 2.7% in the unwounded skin to 11% at seven days post wounding. Thereafter the fraction of PECAM1^+^/Sca1^+^cells declined to 4.8% in 14 days old wounds. Hence, PECAM1^+^/Sca1^+^ cells were enriched at the peak of neoangiogenesis, matrix secretion and myofibroblast appearance.

### PECAM1^+^/Sca1^+^ Vascular Cells Express CD38

**Figure 3:**
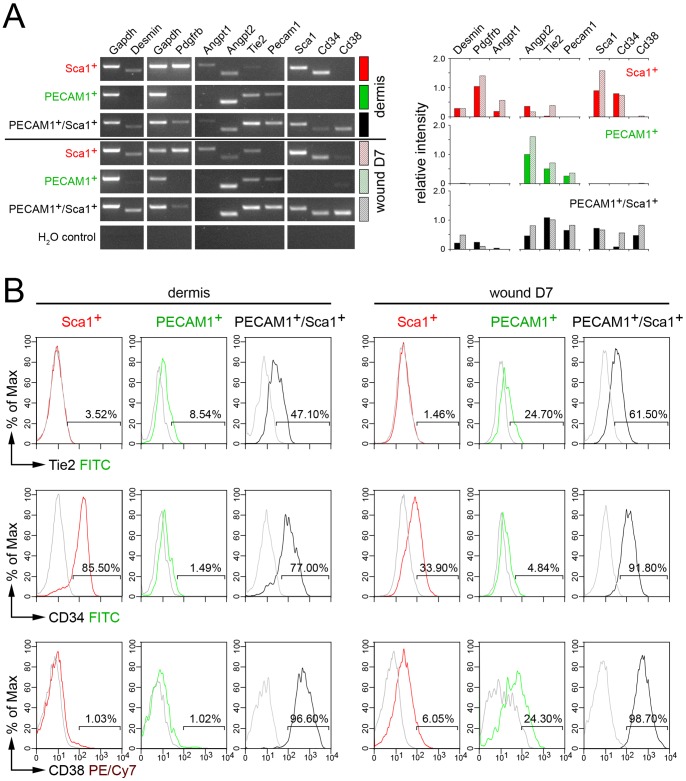
Expression profile of isolated Sca1^+^, PECAM1^+^ and PECAM1^+^/Sca1^+^ **cells.** Marker expression in the sorted cell populations isolated from dermis or wounds seven days post injury. (A) Relative mRNA expression levels of perivascular (*Desmin*, *Pdgfrb*, *Angpt1*), endothelial (*Angpt2*, *Tie2*, *Pecam1*), progenitor cell-specific markers (*Sca1*, *Cd34*) and of the CD38 receptor (*Cd38*) were determined by semiquantitative RT-PCR in non-hematopoietic Sca1^+^, PECAM1^+^ and PECAM1^+^/Sca1^+^ cell populations. The band intensities of the electrophoretic gels were processed and quantified using Image J software. The relative expression intensity of the individual genes compared to *Gapdh* is given. The lack of Sca1 or Pecam1 expression in sorted PECAM1^+^ or Sca1^+^ cells demonstrated the purity of the cell fractions. (B) Flow cytometric detection of TIE2, CD34, CD38 expression at the cell surface of Sca1^+^ (red), PECAM1^+^ (green) and PECAM1^+^/Sca1^+^ (black) cells isolated from dermis or full thickness wounds seven days post injury.

The expression profile of sorted Sca1^+^, PECAM1^+^ and PECAM1^+^/Sca1^+^ cell populations from dermis and wounds seven days post injury were assayed by semiquantitative PCR to define the origin of PECAM1^+^/Sca1^+^ cells (Fig. 3A). Genes expressed in perivascular cells like *Desmin*, *Pdgfrb* and *Angpt1* were upregulated in dermis-derived Sca1^+^ cells, but were hardly detected in PECAM1^+^ cells. These cells mainly expressed endothelial cell-specific genes *Angpt2* and *Tie2*. Surprisingly, perivascular and endothelial cell-specific genes were expressed in PECAM1^+^/Sca1^+^ cells. The progenitor marker *CD34* was detected in Sca1^+^ cells and PECAM1^+^/Sca1^+^ cells and the PECAM1 receptor *CD38* was found exclusively in PECAM1^+^/Sca1^+^ cells. Similar expression profiles were seen in wound-derived cell populations. Flow cytometry confirmed the presence of TIE2, CD34 and CD38 proteins on the cell surface of the distinct cell populations (Fig. 3B). TIE2 protein was missing in Sca1^+^ cells but detected in 9% of PECAM1^+^ and 47% of PECAM1^+^/Sca1^+^ cells of the dermis. Wound-derived Sca1^+^ cells did not express TIE2 protein. The proportion of TIE2-expressing cells increased to 25% for PECAM1^+^ and 62% for PECAM1^+^/Sca1^+^ cells. CD34 was mainly detected on the cell surface of Sca1^+^ and PECAM1^+^/Sca1^+^ cells isolated from dermis or wounds. CD38 protein was found on all dermis-derived PECAM1^+^/Sca1^+^ cells but was not detected on Sca1^+^ and PECAM1^+^ single positive cells. In wounds CD38 expression was maintained on all PECAM1^+^/Sca1^+^ cells and 6% of the Sca1^+^ and 24% of the PECAM1^+^ cells started to express the CD38 receptor. These results indicated that PECAM1^+^/Sca1^+^ cells express CD34 and CD38 protein and activated Sca1^+^ or PECAM1^+^ cells gain CD38 expression upon injury.

**Figure 4:**
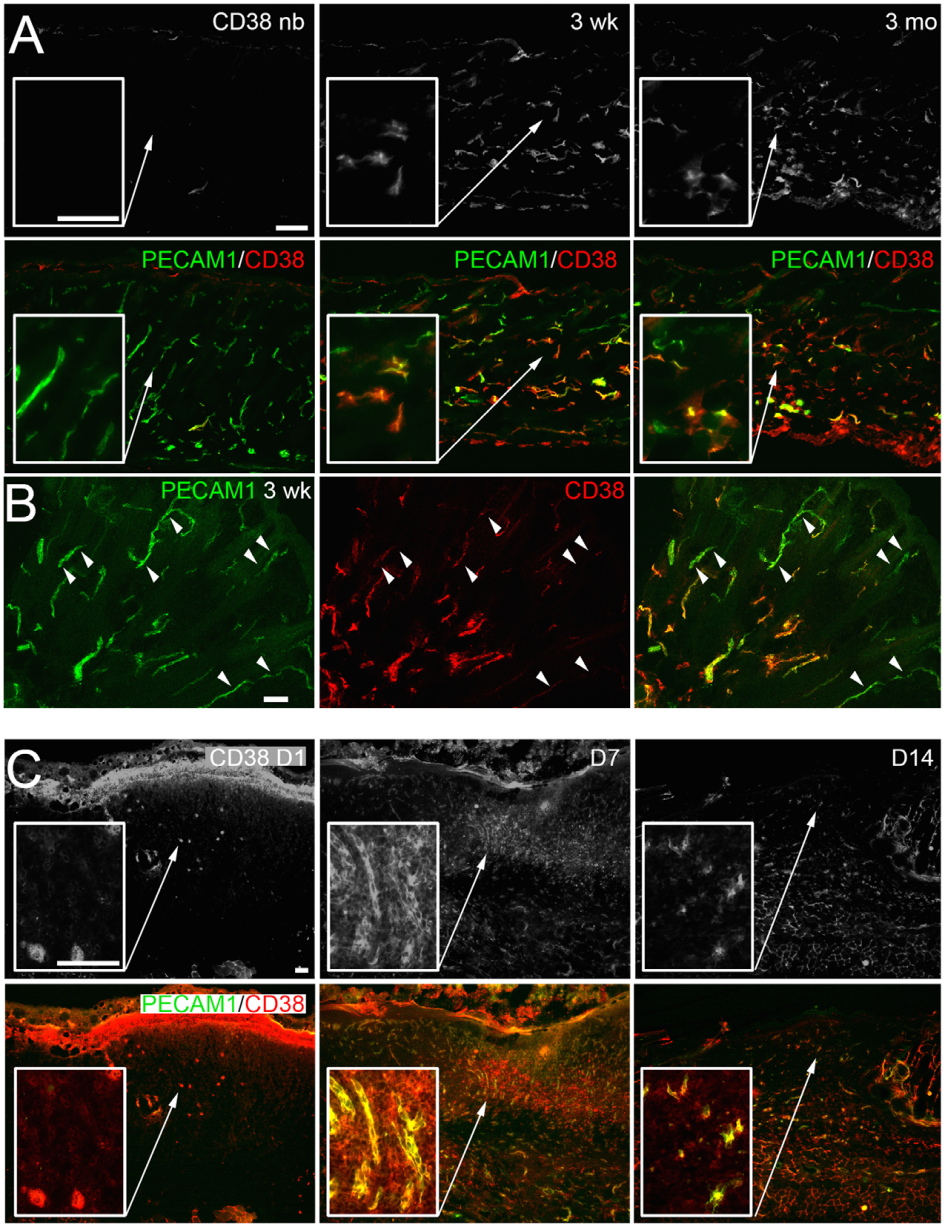
*In situ* detection of the CD38 receptor in the maturing skin and wound. (A) Expression of PECAM1 and CD38 in cryosections of newborn (nb), three weeks (3 wk) and three months (3 mo) old skin was detected by immunofluorescence microscopy. The fluorescence signal for CD38 (top row) and the overlay with PECAM1 is shown. (B) Confocal microscopy analysis of PECAM1/CD38 expression in three weeks old skin. PECAM1^+^ vessels lacking CD38 expression are indicated (arrowheads). (C) Localization of PECAM1 and CD38 expression in cryosections of wounds one (D1), seven (D7) and 14 days (D14) post injury. The fluorescence signal for CD38 (top row), the overlay with PECAM1 and higher magnifications of the wounded area are shown (A, C, squares). Bars 100 µm (A, C), 50 µm (B).

The CD38 receptor mediates cell survival and migration signals [17] and may also activate PECAM1^+^/Sca1^+^ cells in the wound. We therefore studied the localization of CD38^+^ cells in the maturing skin and wounds by immunofluorescence analysis (Fig. 4). In newborn skin no signal for CD38 was seen, although PECAM1^+^ vessels were present throughout the dermis. In contrast, in skin at three weeks and three months a strong signal for CD38 was detected in a subpopulation of PECAM1^+^ vessels of the reticular dermis (Fig. 4A, B). In the early phase of the healing progress (D1) only a few CD38^+^ cells were present in the wound (Fig. 4C), whereas at later stages CD38^+^ cells were found in the intervascular space and in PECAM1^+^ vessels of the granulation tissue (D7). Upon remodeling CD38^+^ cells were mainly associated with PECAM1^+^ vessels (D14). The intervascular distribution of CD38^+^ cells pointed to a role of PECAM1^+^/Sca1^+^/CD38^+^ cells for non-vessel tissue repair.

### PECAM1^+^/Sca1^+^/CD38^+^ Vascular Cells form Myofibroblast-like Cells

**Figure 5:**
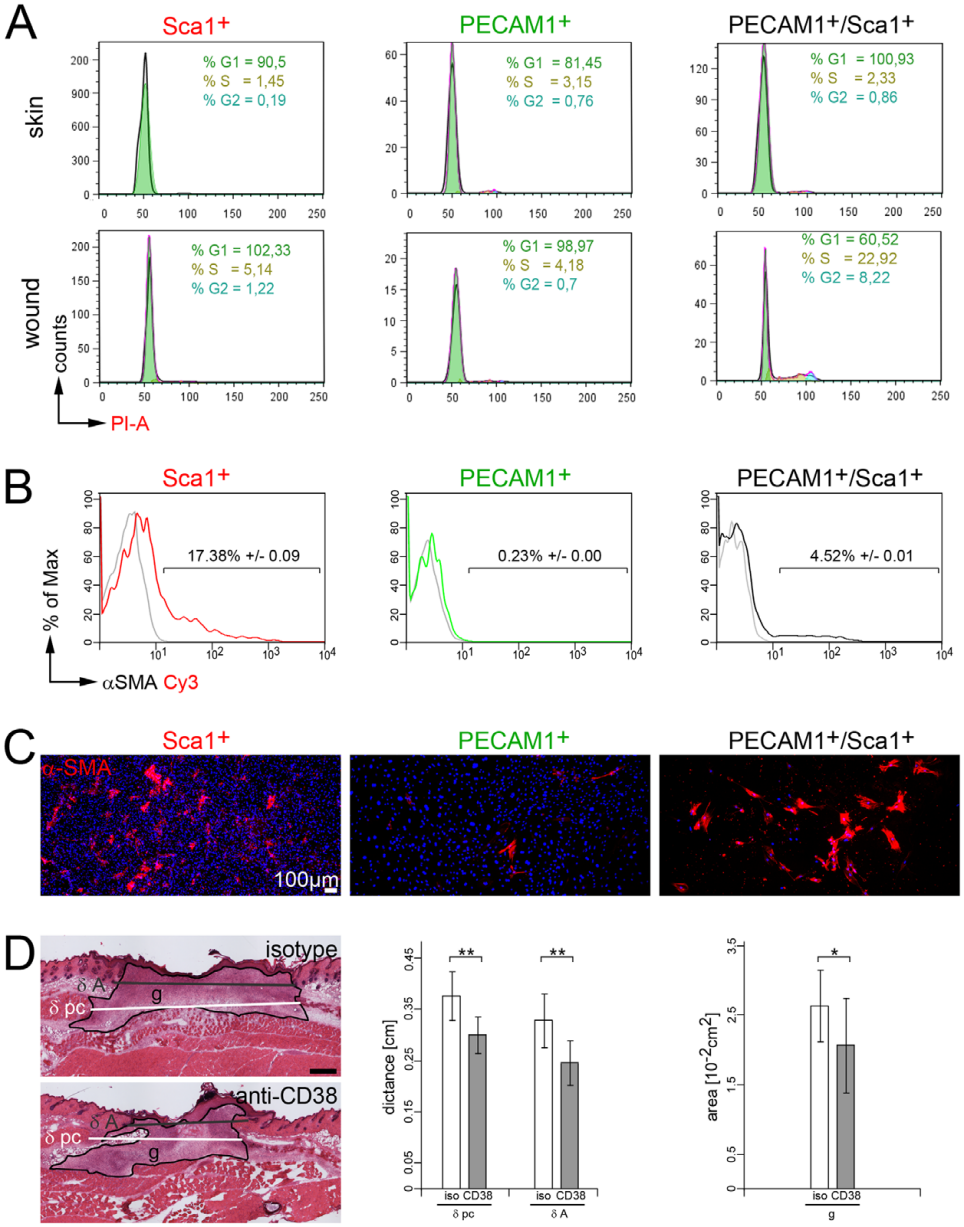
Myofibroblast-like cell formation and modulation of CD38 receptor activity. (A) Representative cell cycle analysis of skin- and wound-derived Sca1^+^, PECAM1^+^ and PECAM1^+^/Sca1^+^ cells seven days post injury using propidium iodide (PI) stain in flow cytometry analysis (see figure S1). The relative percentage of cells in G1 (green), S (ochre) and G2 (blue) are highlighted. (B) Flow cytometric detection of α-SMA in wound-derived Sca1^+^, PECAM1^+^/Sca1^+^ and PECAM1^+^ cells seven days post injury (n = 7 mice). (C) Immunofluorescence analysis of α-SMA expression in cultured wound-derived Sca1^+^, PECAM1^+^ and PECAM1^+^/Sca1^+^ cells. Nucleoli were detected using DAPI stain. (D) Morphometric analysis of wounds in immunodeficient mice stimulated with rat anti-CD38 or isotype matched antibodies (n = 4). Distances between edges of the panniculus carnosus (δ pc), hair follicles (δ A) and the area of the granulation tissue (g) were determined. Statistics: unpaired two-tailed student’s T-test (*p≤0.05, **p≤0.01). Bars 100 µm (C), 500 µm (D).

We speculated that PECAM1^+^/Sca1^+^/CD38^+^ can proliferate and differentiate into myofibroblast-like cells and therefore studied the cell-cycle status and the expression of the myofibroblast marker α-SMA in PECAM1^+^/Sca1^+^/CD38^+^ cells at seven days post wounding (Fig. 5). For cell cycle analysis collagenase-digested skin or wound cell suspensions were stained with Sca1, Pecam1 and the DNA stain propidium iodide and then analyzed by flow cytometry. No proliferation was seen in skin-derived Sca1^+^, PECAM1^+^ or PECAM1^+^/Sca1^+^/CD38^+^ cells. In wounds, most of the Sca1^+^ and PECAM1^+^ were still found in the G_0_/G_1_ phase of the cell cycle, whereas 23% of the PECAM1^+^/Sca1^+^/CD38^+^ cells were found in the S-phase and 8% in the G2 phase of the cell cycle (Fig. 5A, Fig. S1). Hence, only PECAM1^+^/Sca1^+^/CD38^+^ cells proliferate upon wounding at the peak of myofibroblast appearance. In addition, cells of skin and wounds were analyzed for α-SMA expression by flow cytometry analysis. In unwounded skin single PECAM1^+^, Sca1^+^ or PECAM1^+^/Sca1^+^/CD38^+^ cells lack the expression α-SMA (Fig. S2C). This is in line with the immunofluorescence analysis of α-SMA expression in the newborn, 3 weeks and 3 months old skin, where α-SMA could only be found in the glassy membrane of the hair follicles and in the arrector pili muscle muscle tissue, but was nearly absent in the vasculature of the dermis (Fig. S2A). In the wound α-SMA was detected in 17% of Sca1^+^ cells and 4.5% of PECAM1^+^/Sca1^+^ cells (Fig. 5B), whereas PECAM1^+^ cells lack the expression of α-SMA.

In parallel sorted cells were cultured for five days on glass under conditions of mechanical tension and then assessed by immunofluorescence analysis for α-SMA expression. The presence of numerous intact DAPI^+^ nuclei indicated that most cells of the Sca1^+^ or PECAM1^+^ cell population survived the isolation procedure (Fig. 5C), whereas number of PECAM1^+^/Sca1^+^/CD38^+^ cells were significantly reduced. In cultures of Sca1^+^ cells some α-SMA expressing cells emerged, while hardly any α-SMA^+^ cells were present in PECAM1^+^ cell cultures. In contrast, most of the cultured PECAM1^+^/Sca1^+^/CD38^+^ cells expressed α-SMA, displayed a flattened morphology and formed a network of α-SMA^+^ stress fibers. The results indicate that PECAM1^+^/Sca1^+^/CD38^+^ cells can differentiate into α-SMA^+^ myofibroblast-like cells.

To determine the function of CD38 for this differentiation process *in vivo* we triggered the CD38 receptor activity by intravenous injection of a CD38 antibody into wounded mice (n = 4). In order to avoid stimulatory effects of the injected antibody on CD38^+^ lymphoid B- and T-cells, Rag2-deficient mice were used, which have no mature B- or T-cells [18]. Four wounds were inflicted on the back of the mice and the healing progress was studied seven days post injury. Wounds were isolated, sectioned and stained with hematoxylin and eosin (H&E). Morphometric assessment showed a significant reduction in wound size for anti-CD38 antibody-treated mice (Fig. 5D). The distances between the edges of the panniculus carnosus and the hair follicles were shortened by 20% and 25%, respectively, and the granulation tissue was reduced by 21%. These data indicate that CD38 receptor signaling in PECAM1^+^/Sca1^+^ cells promotes vessel-derived myofibroblast formation to support the healing progress.

### PECAM1^+^/CD38^+^ Vascular Cells can be Identified in Human Foreskin and in Vascular Sprouts of Basal Cell Carcinomas

**Figure 6:**
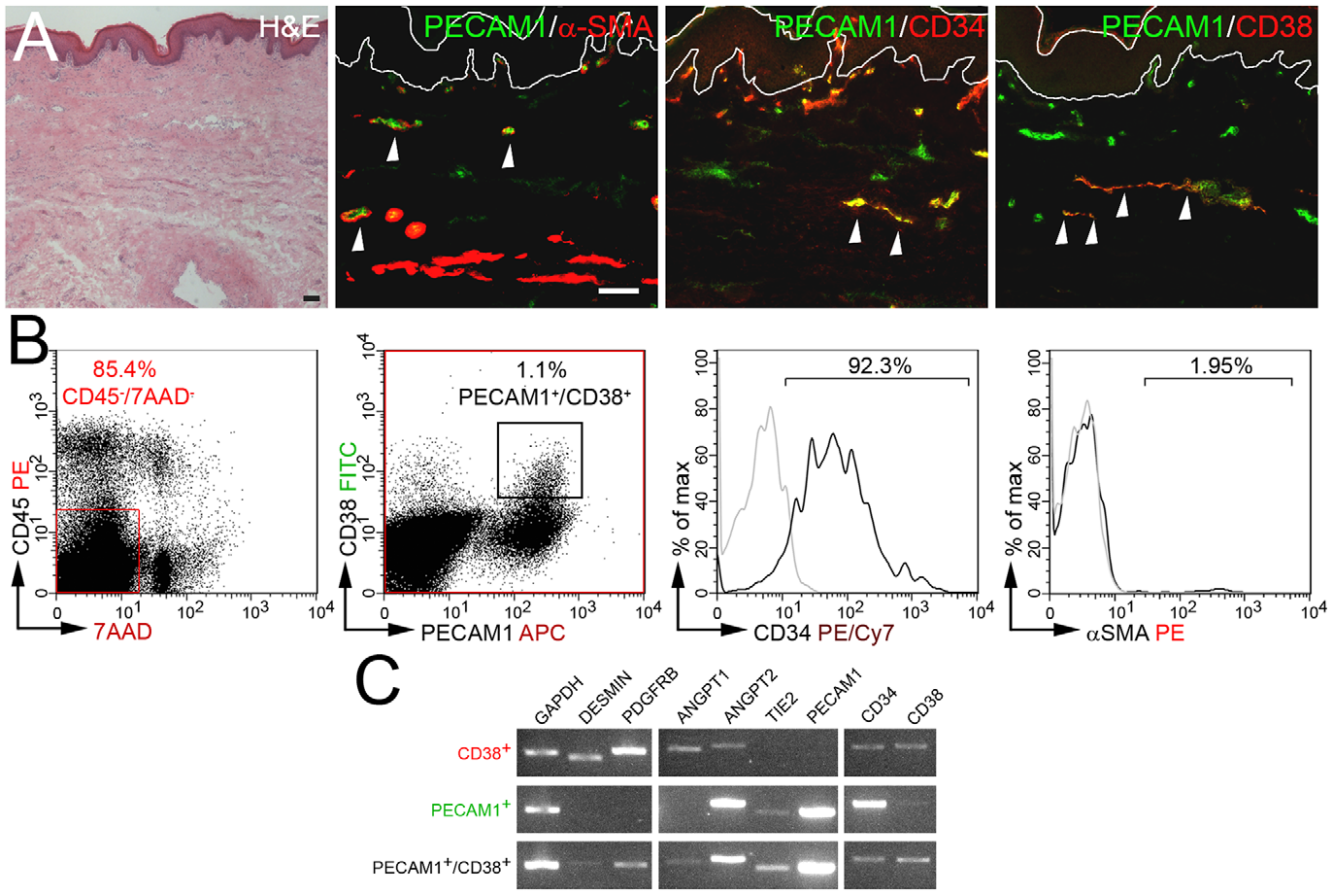
Identification of PECAM1^+^/CD38^+^ cells in human foreskin. (A) Expression of PECAM1, α-SMA, CD34 and CD38 in cryosections of 12 years old foreskin is detected by immunofluorescence microscopy. (B) Flow cytometry analysis of CD38 and PECAM1 expression. The proportion of CD34 and α-SMA expressing cells within the PECAM1^+^/CD38^+^ cell population was determined (histograms). (C) Expression profile of sorted non-hematopoietic CD38^+^, PECAM1^+^ and PECAM1^+^/CD38^+^ cell populations. Bars 100 µm.

To identify a human orthologue vascular cell population we determined the distribution of CD38, PECAM1 and α-SMA by immunofluorescence analysis of human foreskin sections (Fig. 6A). Sca1 was not considered for analysis due to the lack of a human orthologue. PECAM1^+^ endothelial cells were detected throughout the foreskin, whereas only a subpopulation of PECAM1^+^ vessels expressed the CD34 or CD38 receptors. Flow cytometry analysis demonstrated that 1.1% of the viable non-hematopoietic cell population represent PECAM1^+^/CD38^+^ cells and within this population 92% of the cells expressed CD34. Approximately 2% of the PECAM1^+^/CD38^+^ cells contained α-SMA protein as shown by intracellular α-SMA staining (Fig. 6B). In a subsequent semiquantitative PCR study genes preferentially expressed in perivascular cells like *DESMIN*, *PDGFRB* and *ANGPT1* were detected in sorted CD38^+^ cells, whereas endothelial cell-specific genes like *ANGPT2* and *TIE2* were mainly found in sorted PECAM1^+^ cells (Fig. 6C). Both perivascular and endothelial cell-specific genes were expressed in sorted PECAM1^+^/CD38^+^ cells. Hence, a human counterpart for the murine PECAM1^+^/Sca1^+^/CD38^+^ cell population was identified.

**Figure 7:**
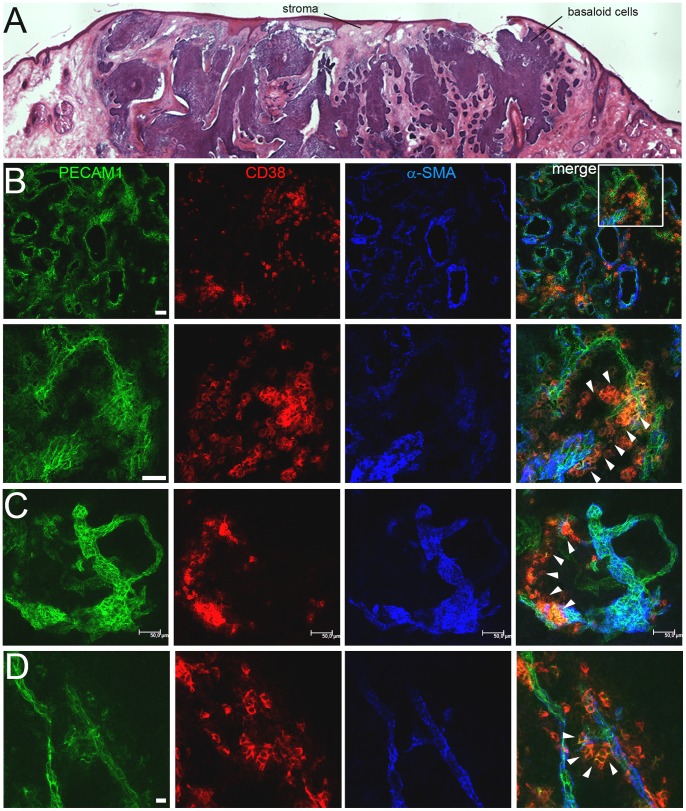
Identification of PECAM1^+^/CD38^+^ cells in human basal cell carcinomas (BCC). (A) H&E-stained cryosection of a BCC. (B–D) Confocal microscopy analysis of PECAM1, CD38 and α-SMA expression in two BCC biopsies (B–C, D). (B) Overview (top row) of the highly vascularized PECAM1^+^ stroma. Squares within the merged image indicate the magnified region (lower row). PECAM1^low^/CD38^+^ cells are marked (arrowheads). Bars 100 µm (A), 50 µm (B, C), 10 µm (D).

The vasculature may contribute to fibrotic tissue formation and pathological neoangiogenesis in humans. Therefore, the distribution of CD38 was studied on cryosections of biopsies from fibrotic scleroderma and neovascularised human basal cell carcinoma (BCC) patients. In four biopsies from patients with late fibrosis no signal for CD38 was detected in immunofluorescent analysis. In contrast, five out of seven BCC biopsies showed a staining for CD38 (Fig. S3). To define CD38^+^ cells sections of two biopsies were costained with antibodies detecting PECAM1, CD38 and α-SMA and analyzed by confocal microscopy. A high density of vessels with strong PECAM1 expression (PECAM1^high^) was found throughout the stroma of the first analyzed biopsy (Fig. 7B). CD38^+^ cells were detected only in areas of low PECAM1 expression (PECAM1^low^) in the outermost PECAM1^+^ cell layer of sprouting vessels. CD38-expressing cells also originated from a dilated vessel to form a PECAM^low^/CD38^+^ loop-like structure (Fig. 7C), whereas the larger PECAM1^high^/α-SMA^+^ vessel and the smaller PECAM1^high^ vessel loop did not contain any CD38^+^ cells. A similar distribution of CD38^+^ cells was seen in the second tumor within a vascular outgrowth from a pre-existing small vessel. CD38 expression was detected in the PECAM1^low^ cells at the vascular tip (Fig. 7D, arrowheads), which seemed to migrate towards the nearby vessel to form a vascular bridge. In summary, CD38^+^ cells are found in neoangiogenic PECAM1^low^ areas of the stromal vasculature in BCC biopsies.

## Discussion

Sca1 and PECAM1 expression is commonly used to identify vascular progenitor cells in various tissues and previous studies suggested that tissue-resident Sca1^+^ [11], [19] or PECAM1^+^/Sca1^+^ [20] precursor cells may exist in the microvasculature to promote neoangiogenesis and tissue repair. In this study PECAM1 and Sca1 expression were used to define the vascular cell populations that are activated during skin repair.

The detection of both markers showed that non-hematopoietic Sca1^+^ and PECAM1^+^/Sca1^+^ are present in the dermis of the skin. Sca1^+^ cells expressed perivascular cell-specific marker genes such as *Pdgfrb* and *Desmin*, but some of the perivascular cell-specific genes may also be expressed by fibroblasts [5]. In contrast, a low proportion of PECAM1^+^/Sca1^+^ cells is found in the skin, which was transiently enriched in the granulation tissue of wounds seven days post injury coinciding temporally with neoangiogenesis and myofibroblast formation. These cells are capable of responding to environmental changes in the wound to enter the cell cycle upon injury and express endothelial and perivascular cell-specific genes as well as the progenitor cell marker CD34. The concomitant expression of endothelial and perivascular cell-specific genes may suggest that endothelial cells transiently undergo endothelial-to-mesenchyme transition in the vasculature of wounded skin. It is remarkable that all cells within the PECAM1^+^/Sca1^+^ population express CD34 and CD38, whereas PECAM1^+^ or Sca1^+^ cells fail to do so. Therefore, the purified cells represent a homogeneous population in a unique localization within the vasculature of the skin. CD34 expression has been reported for bone marrow-derived circulating endothelial progenitors [21]. PECAM1^+^/Sca1^+^/CD38^+^ cells may originate from these CD34^+^ bone marrow-derived precursors but the lack of the hematopoietic CD45 marker expression renders this scenario rather unlikely.

In order to promote non-vessel tissue repair PECAM1^+^/Sca1^+^/CD38^+^ cells have to transform into mesenchymal cells. Myofibroblast-like cells in the intervascular stroma of wounds seven days post injury appear to emerge from pre-existing PECAM1^+^/Sca1^+^/CD38^+^ vessels (see Fig. 1D and Fig. S2B). Mechanical challenge is an important trigger to induce myofibroblast formation from a variety of progenitors [1] and gain of α-SMA expression is a hallmark of this differentiation process. We detected intracellular α-SMA in 4% of the wound-derived PECAM1^+^/Sca1^+^/CD38^+^ cells, which form extended α-SMA containing stress fibres *in vitro*, whereas PECAM1^+^ single positive cells fail to do. Moreover, in cell suspensions from intact skin no intracellular α-SMA was detected in these cell populations. Therefore, it is likely that a proportion of PECAM1^+^/Sca1^+^/CD38^+^ cells can undergo significant phenotypic changes upon wounding to transform into migrating, proliferating and matrix-secreting myofibroblast-like cells to contribute to the pool of myofibroblasts as it was previously described for endothelial cells in cardiac fibrosis [3].

In order to get activated PECAM1^+^/Sca1^+^/CD38^+^ cells need to react to changes in the local environment. CD38 can interact with hyaluronan of the extracellular matrix [22], which is upregulated in skin wounds of mice. Elevated levels of hyaluronan are associated with faster closure, increased tensile strength, and less dermal scarring [23]. We showed that CD38 expression is restricted to the cell surface of PECAM1^+^/Sca1^+^/CD38^+^ cells in the intact and repairing skin. PECAM1^+^/Sca1^+^/CD38^+^ cells in the vasculature may therefore sense changes in the extracellular hyaluronan concentration via the CD38 receptor to become activated.

When stimulating the CD38 receptor by injection of CD38 antibody [24] a pronounced effect on wound healing progression was seen. In these mice the size of the wound was reduced by 20% and although the deposited antibodies hampered the analysis of the distribution of the vasculature we speculate that the stimulation of the PECAM1^+^/Sca1^+^ cell population via the CD38 receptor caused this size reduction. First, CD38 expression is exclusively expressed by PECAM1^+^/Sca1^+^/CD38^+^ cells throughout the healing process (see Fig. 4) and second, effects of anti-CD38 antibodies on CD38^+^ T- or B-cells can be excluded due to the usage of Rag2^−/−^ mice, which fail to produce mature B- or T-lymphocytes [18]. Hence, PECAM1^+^/Sca1^+^/CD38^+^ cells are the most likely target for the crosslinking CD38 antibodies in wounds. Therefore, the reduced wound size could be caused by the altered activation of the CD38 receptor on PECAM1^+^/Sca1^+^ cells, which is accompanied by changes in neoangiogenesis or myofibroblast formation. Interestingly, in human basal cell carcinoma (BCC) PECAM1^low^/CD38^+^ cells are mainly found in the highly vascularized tumor stroma in regions of newly formed vascular sprouts, whereas most of the mature PECAM1^high^ vessels lack CD38 expression. The availability of monoclonal CD38 antibodies [25] and the rather restricted expression upon pathological neoangiogenesis in the human BCCs makes the CD38 receptor also an attractive immunotherapeutic target to prevent new vessel formation and thereby tumor growth.

## Supporting Information

Figure S1 Characterization of the cell proliferation in skin and wound. Cell cycle analysis of skin- and wound-derived Sca1^+^, PECAM1^+^ and PECAM1^+^/Sca1^+^cells seven days post injury using propidium iodide stain in flow cytometry analysis. The histograms of four individual mice are shown (mouse 1–4).
(DOC)

Figure S2 Myofibroblast-like cell formation. (A) Immunostaining of PECAM1/desmin and PECAM1/α-SMA expression at newborn stage (nb), three weeks (3 wk) and three months (3 mo). Squares within the images represent closeups of overlays for the PECAM1/desmin or PECAM1/α-SMA staining. Asterix depict the *arrector pili* muscle and arrows point to the glassy membrane of hair follicles. Only few of the large vessels are surrounded by α-SMA^+^ cells and are highlighted by arrowheads. (B) Immunostaining of PECAM1/α-SMA expression at seven days post injury. The individual monochrome signals for PECAM1 and α-SMA are shown as well as the overlay for the PECAM1/α-SMA staining including the nuclear DAPI staining. PECAM1^+^/α-SMA^+^ cells around PECAM1^+^ vessels are indicated by arrowheads. (C) Flow cytometric detection of α-SMA in Sca1^+^, PECAM1^+^/Sca1^+^ and PECAM1^+^ cells from unwounded skin (n = 7 mice). Bars 100 µm (A), 50 µm (B).
(DOC)

Figure S3 Identification of PECAM1^+^/CD38^+^ cells in vascular sprouts of human basal cell carcinomas. (A) Expression of PECAM1 and CD38 was defined by confocal microscopy analysis of five individual basal cell carcinoma biopsies (patient 1–5). (B) PECAM1 and isotype control (for CD38) staining. Bar 100 µm.
(DOC)

## References

[1] Hinz B (2007) Formation and function of the myofibroblast during tissue repair. J Invest Dermatol 127: 526–537. 17299435 10.1038/sj.jid.5700613

[2] Petroll WM, Barry-Lane PA, Cavanagh HD, Jester JV (1997) ZO-1 reorganization and myofibroblast transformation of corneal endothelial cells after freeze injury in the cat. Exp Eye Res 64: 257–267. 9176060 10.1006/exer.1996.0211

[3] Zeisberg EM, Tarnavski O, Zeisberg M, Dorfman AL, McMullen JR, et al (2007) Endothelial-to-mesenchymal transition contributes to cardiac fibrosis. Nat Med 13: 952–961. 17660828 10.1038/nm1613

[4] Rajkumar VS, Shiwen X, Bostrom M, Leoni P, Muddle J, et al (2006) Platelet-derived growth factor-beta receptor activation is essential for fibroblast and pericyte recruitment during cutaneous wound healing. Am J Pathol 169: 2254–2265. 17148686 10.2353/ajpath.2006.060196 PMC1762470

[5] Eming SA, Brachvogel B, Odorisio T, Koch M (2007) Regulation of angiogenesis: Wound healing as a model. Prog Histochem Cytochem 42: 115–170. 17980716 10.1016/j.proghi.2007.06.001

[6] Sundberg C, Ivarsson M, Gerdin B, Rubin K (1996) Pericytes as collagen-producing cells in excessive dermal scarring. Lab Invest 74: 452–466. 8780163

[7] Brachvogel B, Moch H, Pausch F, Schlotzer-Schrehardt U, Hofmann C, et al (2005) Perivascular cells expressing annexin A5 define a novel mesenchymal stem cell-like population with the capacity to differentiate into multiple mesenchymal lineages. Development 132: 2657–2668. 15857912 10.1242/dev.01846

[8] Goritz C, Dias DO, Tomilin N, Barbacid M, Shupliakov O, et al (2011) A pericyte origin of spinal cord scar tissue. Science 333: 238–242. 21737741 10.1126/science.1203165

[9] Torsney E, Xu Q (2011) Resident vascular progenitor cells. J Mol Cell Cardiol 50: 304–311. 20850452 10.1016/j.yjmcc.2010.09.006

[10] Hoofnagle MH, Wamhoff BR, Owens GK (2004) Lost in transdifferentiation. J Clin Invest 113: 1249–1251. 15124012 10.1172/JCI21761 PMC398438

[11] Grenier G, Scime A, Le Grand F, Asakura A, Perez-Iratxeta C, et al (2007) Resident endothelial precursors in muscle, adipose, and dermis contribute to postnatal vasculogenesis. Stem Cells 25: 3101–3110. 17823241 10.1634/stemcells.2006-0795

[12] Luna G, Paez J, Cardier JE (2004) Expression of the hematopoietic stem cell antigen Sca-1 (LY-6A/E) in liver sinusoidal endothelial cells: possible function of Sca-1 in endothelial cells. Stem Cells Dev 13: 528–535. 15588510 10.1089/scd.2004.13.528

[13] Brachvogel B, Pausch F, Farlie P, Gaipl U, Etich J, et al (2007) Isolated Anxa5(+)/Sca-1(+) perivascular cells from mouse meningeal vasculature retain their perivascular phenotype in vitro and in vivo. Exp Cell Res 313: 2730–2743. 17543301 10.1016/j.yexcr.2007.04.031

[14] Gerharz M, Baranowsky A, Siebolts U, Eming S, Nischt R, et al (2007) Morphometric analysis of murine skin wound healing: standardization of experimental procedures and impact of an advanced multitissue array technique. Wound Repair Regen 15: 105–112. 17244326 10.1111/j.1524-475X.2006.00191.x

[15] Rasband W (1997–2011) ImageJ. U S National Institutes of Health, Bethesda, Maryland, USA.

[16] Watson JV, Chambers SH, Smith PJ (1987) A pragmatic approach to the analysis of DNA histograms with a definable G1 peak. Cytometry 8: 1–8. 3803091 10.1002/cyto.990080101

[17] Deaglio S, Aydin S, Grand MM, Vaisitti T, Bergui L, et al (2010) CD38/CD31 interactions activate genetic pathways leading to proliferation and migration in chronic lymphocytic leukemia cells. Mol Med 16: 87–91. 19956559 10.2119/molmed.2009.00146 PMC2785473

[18] Shinkai Y, Rathbun G, Lam KP, Oltz EM, Stewart V, et al (1992) RAG-2-deficient mice lack mature lymphocytes owing to inability to initiate V(D)J rearrangement. Cell 68: 855–867. 1547487 10.1016/0092-8674(92)90029-c

[19] McQualter JL, Brouard N, Williams B, Baird BN, Sims-Lucas S, et al (2009) Endogenous fibroblastic progenitor cells in the adult mouse lung are highly enriched in the sca-1 positive cell fraction. Stem Cells 27: 623–633. 19074419 10.1634/stemcells.2008-0866

[20] Tsuchiya A, Heike T, Baba S, Fujino H, Umeda K, et al (2008) Sca-1+ endothelial cells (SPECs) reside in the portal area of the liver and contribute to rapid recovery from acute liver disease. Biochem Biophys Res Commun 365: 595–601. 17981147 10.1016/j.bbrc.2007.10.150

[21] Peichev M, Naiyer AJ, Pereira D, Zhu Z, Lane WJ, et al (2000) Expression of VEGFR-2 and AC133 by circulating human CD34(+) cells identifies a population of functional endothelial precursors. Blood 95: 952–958. 10648408

[22] Nishina H, Inageda K, Takahashi K, Hoshino S, Ikeda K, et al (1994) Cell surface antigen CD38 identified as ecto-enzyme of NAD glycohydrolase has hyaluronate-binding activity. Biochem Biophys Res Commun 203: 1318–1323. 8093047 10.1006/bbrc.1994.2326

[23] Mack JA, Abramson SR, Ben Y, Coffin JC, Rothrock JK, et al (2003) Hoxb13 knockout adult skin exhibits high levels of hyaluronan and enhanced wound healing. Faseb J 17: 1352–1354. 12759339 10.1096/fj.02-0959fje

[24] Hara-Yokoyama M, Kimura T, Kaku H, Wakiyama M, Kaitsu Y, et al (2008) Alteration of enzymatic properties of cell-surface antigen CD38 by agonistic anti-CD38 antibodies that prolong B cell survival and induce activation. Int Immunopharmacol 8: 59–70. 18068101 10.1016/j.intimp.2007.10.010

[25] de Weers M, Tai YT, van der Veer MS, Bakker JM, Vink T, et al (2011) Daratumumab, a novel therapeutic human CD38 monoclonal antibody, induces killing of multiple myeloma and other hematological tumors. J Immunol 186: 1840–1848. 21187443 10.4049/jimmunol.1003032

