# Supplementary material for: PECAM1+/Sca1+/CD38+ Vascular Cells Transform into Myofibroblast-Like Cells in Skin Wound Repair
Source: PLoS One. 2013 Jan 4;8(1):e53262. doi: 10.1371/journal.pone.0053262 (PMC3537615; doi:10.1371/journal.pone.0053262)
Supplement: Figure S1 — Characterization of the cell proliferation in skin and wound. Cell cycle analysis of skin- and wound-derived Sca1+, PECAM1+ and PECAM1+/Sca1+cells seven days post injury using propidium iodide stain in flow cytometry analysis. The histograms of four individual mice are shown (mouse 1–4). (DOC) [file pone.0053262.s001.doc]

**Supporting information**


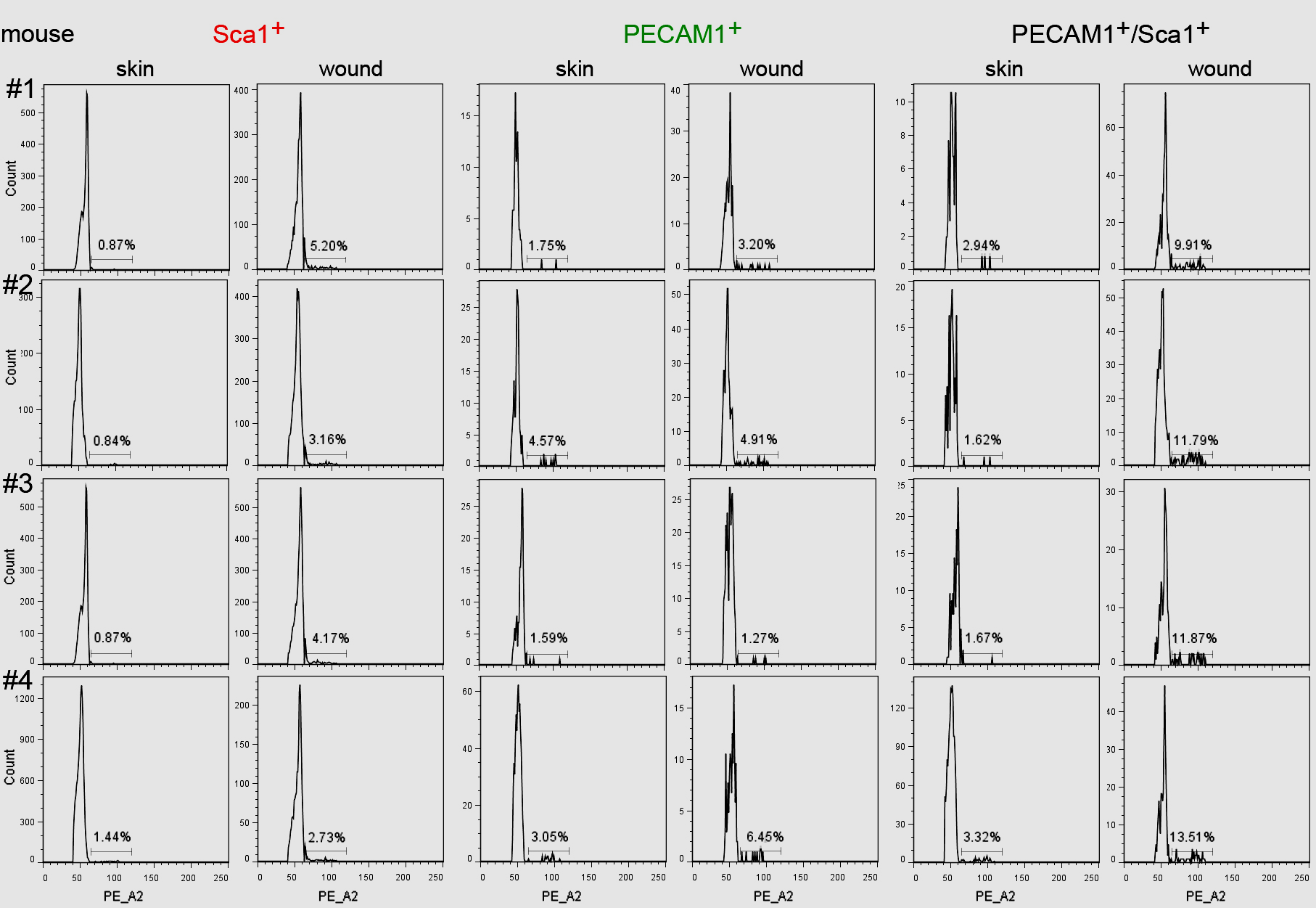


**Figure S1. Characterization of the cell proliferation in skin and wound.** Cell cycle analysis of skin- and wound-derived Sca1^+^, PECAM1^+^/Sca1^+^ and PECAM1^+^ cells seven days post injury using propidium iodide stain in flow cytometry analysis. The histograms of four individual mice are shown (mouse 1-4).
