# Supplementary material for: PECAM1+/Sca1+/CD38+ Vascular Cells Transform into Myofibroblast-Like Cells in Skin Wound Repair
Source: PLoS One. 2013 Jan 4;8(1):e53262. doi: 10.1371/journal.pone.0053262 (PMC3537615; doi:10.1371/journal.pone.0053262)
Supplement: Figure S2 — Myofibroblast-like cell formation. (A) Immunostaining of PECAM1/desmin and PECAM1/α-SMA expression at newborn stage (nb), three weeks (3 wk) and three months (3 mo). Squares within the images represent closeups of overlays for the PECAM1/desmin or PECAM1/α-SMA staining. Asterix depict the arrector pili muscle and arrows point to the glassy membrane of hair follicles. Only few of the large vessels are surrounded by α-SMA+ cells and are highlighted by arrowheads. (B) Immunostaining of PECAM1/α-SMA expression at seven days post injury. The individual monochrome signals for PECAM1 and α-SMA are shown as well as the overlay for the PECAM1/α-SMA staining including the nuclear DAPI staining. PECAM1+/α-SMA+ cells around PECAM1+ vessels are indicated by arrowheads. (C) Flow cytometric detection of α-SMA in Sca1+, PECAM1+/Sca1+ and PECAM1+ cells from unwounded skin (n = 7 mice). Bars 100 µm (A), 50 µm (B). (DOC) [file pone.0053262.s002.doc]

**
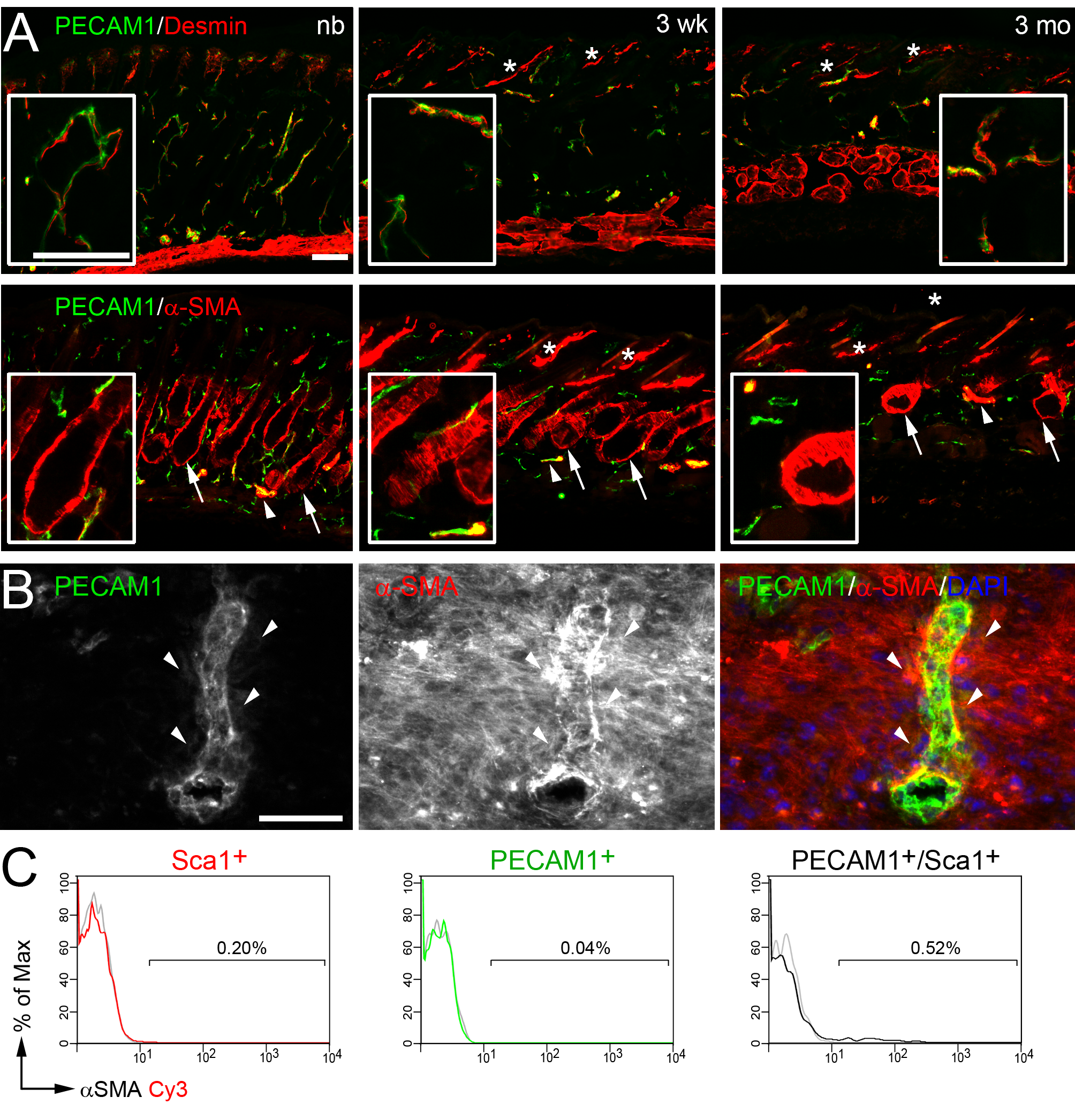
**

**Figure S2. Myofibroblast-like cell formation.** (A) Immunostaining of PECAM1/desmin and PECAM1/α-SMA expression at newborn stage (nb), three weeks (3 wk) and three months (3 mo). Squares within the images represent closeups of overlays for the PECAM1/desmin or PECAM1/α-SMA staining. Asterix depict the *arrector pili* muscle and arrows point to the glassy membrane of hair follicles. Only few of the large vessels are surrounded by α-SMA^+^ cells and are highlighted by arrowheads. (B) Immunostaining of PECAM1/α-SMA expression at seven days post injury. The individual monochrome signals for PECAM1 and α-SMA are shown as well as the overlay for the PECAM1/α-SMA staining including the nuclear DAPI staining. PECAM1^+^/α-SMA^+^ cells around PECAM1^+^ vessels are indicated by arrowheads. (C) Flow cytometric detection of α-SMA in Sca1^+^, PECAM1^+^/Sca1^+^ and PECAM1^+^ cells from unwounded skin (n=7 mice). Bars 100 µm (A), 50 µm (B).
