# Supplementary material for: PECAM1+/Sca1+/CD38+ Vascular Cells Transform into Myofibroblast-Like Cells in Skin Wound Repair
Source: PLoS One. 2013 Jan 4;8(1):e53262. doi: 10.1371/journal.pone.0053262 (PMC3537615; doi:10.1371/journal.pone.0053262)
Supplement: Figure S3 — Identification of PECAM1+/CD38+ cells in vascular sprouts of human basal cell carcinomas. (A) Expression of PECAM1 and CD38 was defined by confocal microscopy analysis of five individual basal cell carcinoma biopsies (patient 1–5). (B) PECAM1 and isotype control (for CD38) staining. Bar 100 µm. (DOC) [file pone.0053262.s003.doc]

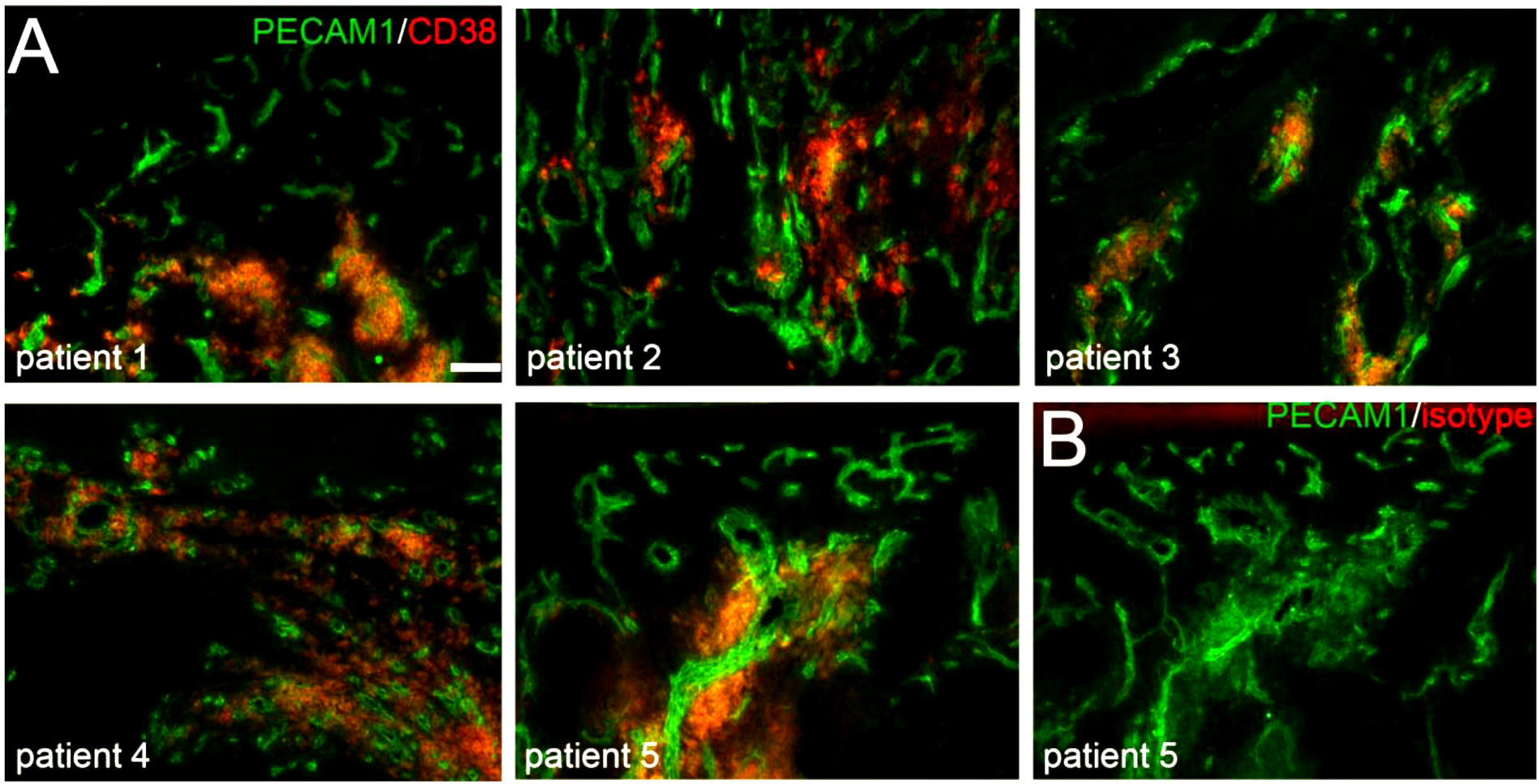


**Figure S3. Identification ofPECAM1^+^/CD38^+^ cells in vascular sprouts of human basal cell carcinomas.** (A) Expression of PECAM1 and CD38 was defined by confocal microscopy analysis of five individual basal cell carcinoma biopsies (patient 1-5). (B) PECAM1 and isotype control (for CD38) staining. Bar 100µm.
